# Coded DNN Watermark: Robustness against Pruning Models Using Constant Weight Code

**DOI:** 10.3390/jimaging8060152

**Published:** 2022-05-26

**Authors:** Tatsuya Yasui, Takuro Tanaka, Asad Malik, Minoru Kuribayashi

**Affiliations:** 1 Graduate School of Natural Science and Technology, Okayama University, Okayama 700-8530, Japan; pd3x1vnt@s.okayama-u.ac.jp (T.T.); kminoru@okayama-u.ac.jp (M.K.); 2 Department of Computer Science, Aligarh Muslim University, Aligarh 202002, India; amalik_co@myamu.ac.in

**Keywords:** watermarking, pruning attack, DNN model, constant weight code, fine-tuning

## Abstract

Deep Neural Network (DNN) watermarking techniques are increasingly being used to protect the intellectual property of DNN models. Basically, DNN watermarking is a technique to insert side information into the DNN model without significantly degrading the performance of its original task. A pruning attack is a threat to DNN watermarking, wherein the less important neurons in the model are pruned to make it faster and more compact. As a result, removing the watermark from the DNN model is possible. This study investigates a channel coding approach to protect DNN watermarking against pruning attacks. The channel model differs completely from conventional models involving digital images. Determining the suitable encoding methods for DNN watermarking remains an open problem. Herein, we presented a novel encoding approach using constant weight codes to protect the DNN watermarking against pruning attacks. The experimental results confirmed that the robustness against pruning attacks could be controlled by carefully setting two thresholds for binary symbols in the codeword.

## 1. Introduction

Digital watermarking has been studied for a long time to preserve the copyright of digital data such as image, audio, and video by inserting some confidential information. In addition, the widespread use of Deep Neural Network (DNN) models in the current scenario is crucial to protect their copyrights. Researchers have been studying DNN watermarking to protect the intellectual property associated with DNN models. Because of the multiple network layers in a DNN model, many parameters known as network weights must be trained to attain a local minimum. However, several degrees of freedom are available for choosing the weight parameters for embedding a watermark. Moreover, the watermark is inserted in such a way that the accuracy of the model on its original task decreases to the lowest extent possible.

DNN watermarking techniques can be categorized into two types [1]: white-box watermarking, black-box watermarking. In white-box watermarking, internal architecture and parameters are exposed to the public, and black-box watermarking takes advantage of the functionality of DNN models. In some cases, when a specific query is input, the watermark can be retrieved from its output without knowing the internal parameters; this characteristic is equivalent to creating a backdoor into the model. Basically, in the black-box methodology can only access the final layer’s output, some experts have investigated training networks to intentionally make wrong output for a given input and then use it as a watermark [2,3]. Moreover, the research into black-box watermarking is also receiving a great deal of attention, especially in the frequency domain [4,5], which performs well in terms of imperceptibility and robustness.

The first white-box method was presented in [6,7], where a watermark was embedded into the weight parameters of a convolutional neural network (CNN) model. The embedding operation is performed simultaneously along with the training of the model by introducing an embedding loss function so that the weights are updated according to the watermark and the supervised dataset.

In [8,9], the selection of the feature vector in these methods presented in [6,7] was refined. In [10,11] reported that almost all local minima are very similar to the global optimum. Empirical evidence has shown that a local minimum for deeper or larger models is sufficient because their loss values are similar. With this characteristic, the watermark was embedded into some sampled weight values in [12,13]. In [13], the sample weight values were inputted to a DNN model that is independent of the host model, and error back-propagation was used to embed the watermark in both the host model and the independent model. The white-box method must be sufficiently robust to recover the watermark from a perturbed version of a DNN model because attackers can directly modify the parameters. One instance of perturbation is model pruning, where redundant neurons are pruned without compromising accuracy to reduce the computational costs of executing DNN models. The purpose of pruning is to remove less-important weight parameters from the DNN model whose contribution to the loss is small. If the watermark signal is embedded into such less-important parameters, it is easily removed or modified by pruning. Therefore, a crucial requirement of DNN watermarking is the robustness against pruning attacks [14] while ensuring that the watermarked parameters are relevant to the original task. Uchida et al. showed experimentally that the watermark does not disappear even after a pruning attack that prunes 65% of the parameters [6]. Another study achieved robustness against 60% pruning [15]. This study adopted the idea of spread transform dither modulation (ST-DM) watermarking by extending the conventional spread spectrum (SS)-based DNN watermarking. A detailed survey of DNN watermarking techniques can be summarized in [16].

Another study in [17], embedding the watermark into the model structure by pruning has been proposed.This method was shown to be robust against attacks that adjust the model’s weights, which is a threat in other embedding methods. Moreover, this method considers pruning an embedding method, whereas we consider pruning as a perturbation by the attacker and propose a robust embedding method against pruning. In communication channels, pruning can be regarded as an erasure channel between the transmitter and the receiver of the watermark. Because numerous symbols are erased over the channel (e.g., more than half of the weight parameters are erased by pruning), erasure correcting codes are unsuitable for this channel.

In this study, we encode the watermark using binary constant weight codes (CWC) [18,19] to make it robust against weight-level pruning attacks. The preliminary version of this paper is available at [20]. The symbols “1” used in the codeword are fixed and designed to be as small as possible. Thus, most of the symbols used in the codeword are becoming ”0”. To embed such a codeword, we enforce a constraint by using two thresholds while training the DNN model. The amplitude for symbol “1” is controlled to be greater than a high threshold, and that for symbol “0” is controlled to be smaller than a low threshold. Once a pruning attack is executed, the erasure of weight parameters does not affect the symbols “0” because these symbols can be extracted even if the amplitude is small. On the other hand, the symbol “1” can be detected correctly because of the high amplitude. Under the assumption that the values of weight parameters follow Gaussian or uniform distribution, the design of the two thresholds is considered to ensure robustness against pruning attacks. In the experiment, we evaluate validity of the thresholds in terms of pruning attacks and retraining of the pruned models.

Our contribution is the introduction of encoding technique into the DNN watermark to make it robust against pruning attacks. While previous studies have proposed DNN watermarks that are robust against a certain level of pruning rate, our method can assure the robustness with a pre-defined level of pruning rate by carefully setting the encoding parameters. The common scenario in which DNN watermarks are used in a DNN model is the buying and selling of DNN models. In this scenario, our method can prevent illegal redistribution and illegal copying by users who have purchased the DNN model. As a white-box watermarking is assumed in our method, it suffers from the direct modification of weight parameters, which is a common threat in white-box settings. If the weight parameters are replaced with random values and trained from scratch with a sufficient dataset, the watermark can be removed completely without compromising performance of the DNN model. Hence, it is assumed in our method that the attacker cannot train the target DNN model from scratch in terms of computational resources and training dataset.

The remainder of this paper is organized as follows. Section 2 presents some assumptions of parameters. The proposed method is detailed in Section 3, and experimental results are presented in Section 4. Finally, conclusions are drawn in Section 5, followed by suggestions for future work.

## 2. Preliminaries

### 2.1. Notation

The notation of parameters used in this paper are summarized below:

- *N*: Number of weight parameters in DNN model.
- *L*: Number of selected weight parameters.
- $w=\left(w_{0},w_{1},\ldots ,w_{L-1}\right),w_{i}\in R$: Selected weight parameters.
- $w^{\prime }$: Selected weight parameters after pruning attack.
- *R*: Pruning rate.
- p=⌊RN⌋: Number of pruned weight parameters.
- *k*: Bit length of watermark.
- $b=\left(b_{0},b_{1},\ldots ,b_{k-1}\right),b_{i}\in \left\{0,1\right\}$: Binary watermark.
- $c=\left(c_{0},c_{1},\ldots ,c_{L-1}\right),c_{i}\in \left\{0,1\right\}$: Binary codeword of CWC.
- $\alpha =\sum c_{i}$: Hamming weight of codewords.
- $T_{1}$, $T_{0}$: Thresholds for binary classification, where $0<T_{0}<T_{1}$.
- sort(): Sort algorithm with ascending order.
- sgn(): Sign function.

### 2.2. Pruning DNN Model

Owing to the many neurons in DNN models, there is significant redundancy in the network; making a network deeper is a promising way to improve the performance. It is reasonable to prune such redundant neurons to reduce the model size as well as to reduce computational costs [21]. It is an NP-hard problem to find the best combination of neurons to be pruned, from among the millions of parameters in a DNN model [22]. Some heuristic pruning methods have been developed to identify relatively less important components in DNN models and retrain the pruned model to recover the model’s performance. Thus, to create a robust DNN watermark, it is necessary to consider the effects of pruning as well as the changes during retraining.

The pruning methods can be roughly classified into three categories. One is weight-level pruning, which sets less important weights to zero and does not change the network structure. The other two are channel-level and layer-level pruning, which can change the network structure but require large computations to find an efficient network modification with little compromise in performance. Therefore, we focus on weight-level pruning in this study. In the weight-level pruning, after training, the parameters whose absolute values are lower than a threshold are cut-off to zero to compress the DNN model. The threshold is set such that the model’s accuracy does not decrease significantly.

For a given rate 0≤R<1, the pruning attack changes the weight values $w_{i}=0$ if $|w_{i}|<{\tilde{w}}_{p}$ for 0≤i≤N−1, where

(1) $$\tilde{w}=sort\left(|w|\right)=sort(|w_{0}\left|,\right|w_{1}\left|,\ldots ,\right|w_{N-1}\left|\right),$$

sort() is a sort algorithm, and

(2) p=⌊RN⌋.

Thus, according to the rate *R*, the weight parameters whose absolute values are smaller than the *p*-th weight are pruned.

Han et al. [23] proposed to prune network weights with small magnitude by incorporating a deep compression pipeline to create efficient models. Some criteria and settings have been proposed for weight-level pruning [24,25]. Some types of weight-level pruning can be viewed as a process to find a mask to determine which weights to preserve.

### 2.3. Constant Weight Code

CWC C(α,L) with parameters α and *L* is a set of binary codewords of length *L*, all having weight α; it has a fixed Hamming weight. Therefore, a codeword $c=\left(c_{0},c_{1},\ldots ,c_{L-1}\right),c_{i}\in \left\{0,1\right\}$ of CWC satisfies the condition such that

(3) $$\sum_{i=0}^{L-1}c_{i}=\alpha ,$$

where α is fixed constant.

It is clear that no two codewords in C(α,L) have Hamming distance 1 and the minimum distance is 2. This means that the code can detect only a single error and cannot correct any error at all. To make the CWC more practical, some researchers have been involved in developing a code with the restriction of a minimum distance *d*. The main problem in coding theory is finding the maximum possible number of codewords in a CWC with length *L*, minimum distance *d*, and weight α. Such CWCs have been extensively studied by MacWilliams and Sloane [26]. A large table of lower bounds on these numbers was published by Brouwer et al. [19], and it was updated by Smith et al. [27]. Because the CWC has no error-correction capability, even a 1-bit error is not allowed. Some studies have investigated CWCs with error-correcting capabilities [28,29,30]. These codes are a promising way to enhance the robustness against other attacks for DNN watermarking. Because of the simplicity, in our approach, we have adopted Schalkwijk’s algorithm [18] for encoding and decoding the CWC with minimum distance 2, which does not restrict the use of other algorithms for constant weight code.

The encoding algorithm in [18] maps *k*-bit information into a codeword *c* with weight α and length *L*. The procedure to encode ***b*** into a codeword c∈C(α,L) is described in Algorithm 1. The *k*-bit information ***b*** is recovered by decoding the codeword *c* using Algorithm 2, where “≫” denotes the right bit-shift operator.

**Algorithm 1** Encode ***b*** into ***c*****Input:**  α, *L*, $b=(b_{0},b_{1},\ldots ,b_{k-1})$, $b_{t}\in \left\{0,1\right\}$**Output:**  $c=(c_{0},c_{1},\ldots ,c_{L-1})$, $c_{t}\in \left\{0,1\right\}$  1:  $B\leftarrow \sum_{t=0}^{k-1}b_{t}2^{t}$;  2:  l←α;  3:  **for** 
t=0 **to** 
L−1 
**do**  4:    **if**
B≥L−t−1l
**then**  5:     $c_{L-t-1}=1$;  6:     B←B−L−t−1l;  7:     l←l−1;  8:   **else**  9:     $c_{L-t-1}=0$;10:   **end if**11: **end for**


**Algorithm 2** Decode ***c*** into ***b*****Input:**  
α, *L*, $c=(c_{0},c_{1},\ldots ,c_{L-1})$, $c_{t}\in \left\{0,1\right\}$
**Output:**  
$b=(b_{0},b_{1},\ldots ,b_{k-1})$, $b_{t}\in \left\{0,1\right\}$  1:  
B←0;  2:  l←0;  3:  **for** 
t=0 **to** 
L−1 
**do**  4:   **if** $c_{t}=1$ **then**  5:     l←l+1;  6:     B←B+tl;  7:   **end if**  8: **end for**  9: **for** 
t=0 to
k−1 
**do**10:   $b_{t}=B\;\left(\mathrm{mod}\;2\right)$11:   B←B≫1;12:  **end for**

## 3. Proposed DNN Watermarking

**Figure 1:**
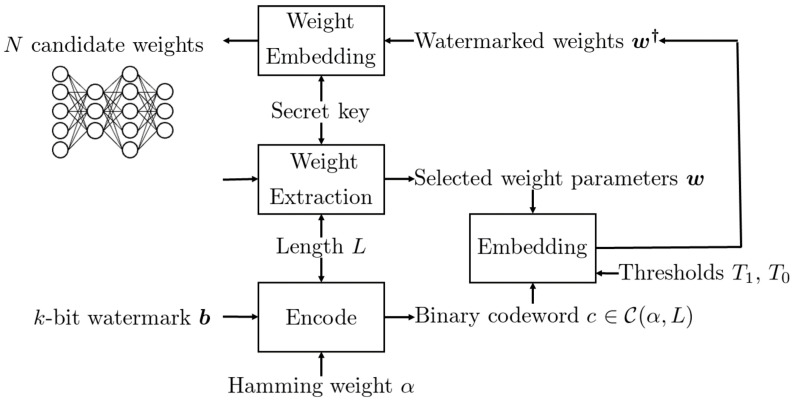
Flow diagram of embedding procedure.

**Figure 2:**
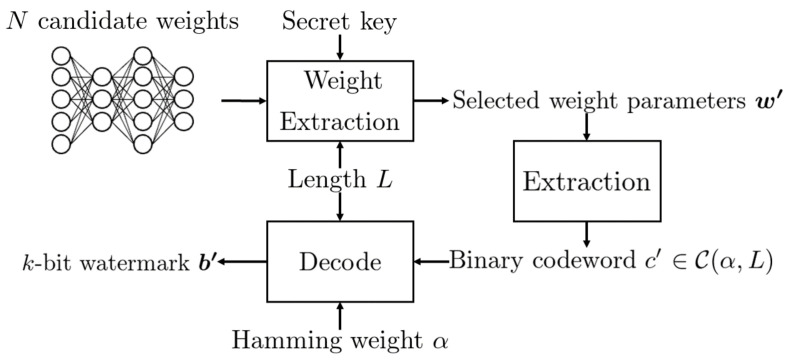
Flow diagram of extraction procedure.

The overview of the proposed DNN watermarking is shown in Figure 1 and Figure 2, where Figure 1 represents the embedding procedure and Figure 2 represents the extraction procedure.

The idea is to encode the *k*-bit watermark ***b*** into the codeword *c* using CWC before the embedding operation. The weights corresponding to the elements $c_{i}=1$ become more than a higher threshold $T_{1}$, while the others corresponding to $c_{i}=0$ becomes less than a lower threshold $T_{0}$ by the embedding operation. In case the pruning attack is executed to round weight parameters $w_{i}$ with a small value to 0, those elements are judged as bit 0 in the codeword, and hence, there is no effect on the received codeword. As for bit 1, the corresponding weight parameters $w_{i}$ should be sufficiently large so that these are not cut off.

During initialization of a given DNN model, *L* weight parameters ***w*** are selected from *N* candidates according to a secret key. Then, an encoded watermark ***c*** is embedded into ***w*** under the following constraint:

- If $c_{i}=1$, then $|w_{i}|\geq T_{1}$; otherwise, $|w_{i}|\leq T_{0}$, where $T_{0}$ and $T_{1}$ are thresholds satisfying $0<T_{0}<T_{1}$.

In the training process of a DNN model, weight parameters are updated iteratively to converge into a local minimum. The changes to the weights ***w*** selected for embedding ***c*** are only controlled by the above restriction during the training process in the proposed method.

### 3.1. Embedding

First, we encode a *k*-bit watermark ***b*** into the codeword ***c*** by using Algorithm 1. Here, the parameters α and *L* must satisfy the following condition:

(4) 2k≤Lα=L!α!(L−α)!<2k+1.

During the embedding operation, the weight parameters ***w*** selected from the DNN model are modified into $w^{†}=\left(w_{0}^{†},w_{1}^{†},\ldots ,w_{L-1}^{†}\right)$ by using the two thresholds $T_{1}$ and $T_{0}$.

(5) $$w_{i}^{†}=\left\{\begin{array}{cc} w_{i} & \left(c_{i}=1\right)\cap \left(\right|w_{i}\left|\geq T_{1}\right)\\ sgn\left(w_{i}\right)\times T_{1} & \left(c_{i}=1\right)\cap \left(\right|w_{i}\left|<T_{1}\right)\\ w_{i} & \left(c_{i}=0\right)\cap \left(\right|w_{i}\left|\leq T_{0}\right)\\ sgn\left(w_{i}\right)\times T_{0} & \left(c_{i}=0\right)\cap \left(\right|w_{i}\left|>T_{0}\right) \end{array}\right.,$$

where

(6) $$sgn\left(x\right)=\left\{\begin{array}{cc} 1 & x\geq 0\\ -1 & x<0 \end{array}\right.$$

**Figure 3:**
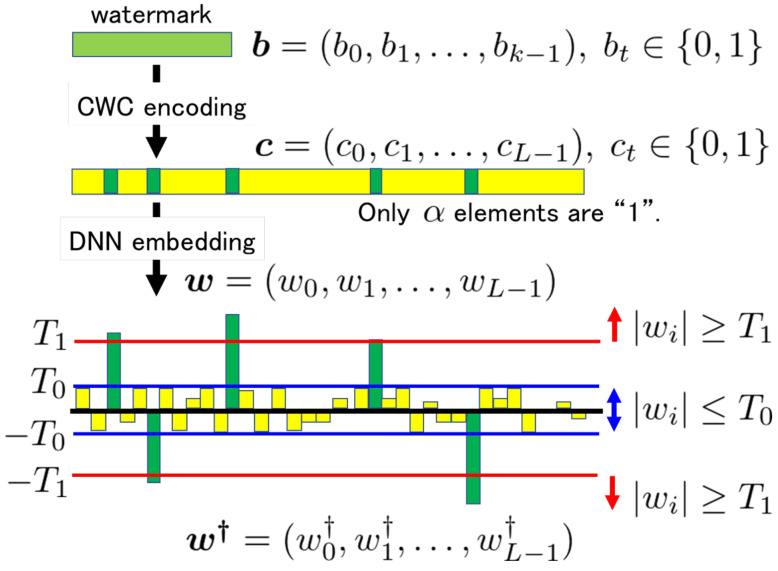
Illustration of embedding procedure.

The embedding procedure is illustrated in Figure 3.

Equation (5) can be regarded as a constraint for executing the training process for the DNN model to embed the watermark. Among the numerous *N* candidates, we impose the constraint only on the *L* weight parameters selected for embedding.

### 3.2. Extraction

It is expected that the distribution of selected weights to be embedded is the same as the distribution of all candidates (Gaussian or uniform distribution). When embedding a watermark, the change of the distribution depends on the thresholds $T_{1}$ and $T_{0}$ as well as the length *L* of encoded watermark.

#### Detection

To check the presence of watermark in a DNN model, it is sufficient to check the bias in the distribution of selected *L* weights. Here, if the secret key for selecting weights is known, the bias of the binary sequence of the selected weights can be used for checking the existence of watermark. Because the red Hamming weight of the binary sequence is α and it is much smaller than *L*, the bias is useful to detect the existence of watermark. Under the assumption is that *L* is extremely small compared with all candidates, it is difficult, without the secret key, to find and change the selected weights under the constraint that the performance degradation of the watermarked model is negligibly small.

If all weights in the DNN model are uniformly distributed, then the randomly selected weights are also uniformly distributed. To check whether the sequence of selected weights is the CWC codeword or not, we measure the mean square error (MSE) of the sequence. Suppose that the weights are selected from a uniform distribution with range [0,δ]. If the sequence of selected weights is not a CWC codeword, the distribution is the uniform distribution in the range [0,δ] and the mean is δ/2. On the other hand, if a CWC codeword is embedded, the distribution is different and is dependent on $T_{1}$ and $T_{0}$ as follows:

- Top α-th weights: the uniform distribution in the range $\left[T_{1},\delta \right]$, the mean is $(\delta +T_{1})/2$.
- Remainder: the uniform distribution in the range $\left[0,T_{0}\right]$, the mean is $T_{0}/2$.

We can determine whether the distribution of the sequence of weights is similar to the CWC codeword or not. The MSE per the metrics is defined by the following equation.

(7) $$MSE=\frac{1}{L}\sum_{i=0}^{L-1}d_{i},$$

where

(8) $$d_{i}=\left\{\begin{array}{cc} {\left(c_{i}-\frac{\delta +T_{1}}{2}\right)}^{2} & \left(1\leq i<\alpha \right)\\ {\left(c_{i}-\frac{T_{0}}{2}\right)}^{2} & \left(\alpha \leq i<L\right) \end{array}\right..$$

### 3.3. Recovery of Watermark

First, the weight parameters are selected from the same DNN model positions, denoted by $w^{\prime }$. Then, the α-th largest element ${\tilde{w}}_{L-\alpha }^{\prime }$ is determined from $w^{\prime }$, and the codeword $c^{\prime }$ is constructed as follows:

(9) $$c_{i}^{\prime }=\left\{\begin{array}{cc} 1\; & \;if\;|w_{i}^{\prime }|\geq {\tilde{w}}_{L-\alpha }^{\prime }\\ 0\; & \;otherwise \end{array}\right.,$$

where ${\tilde{w}}^{\prime }=sort(|w^{\prime }\left|\right)$. Finally, using Algorithm 2, the watermark $b^{\prime }$ is reconstructed from the codeword $c^{\prime }$ as the result.

In the above operation, the top-α symbols in $w^{\prime }$ are regarded as “1”, and the others are “0”. Even if L−α symbols whose absolute value is smaller than that of the top-α symbols are pruned, the codeword can be correctly reconstructed from the weight parameters $w^{\prime }$ in the pruned DNN model. When the pruning rate *R* satisfies the condition

(10) $$R<\frac{L-\alpha }{L}=\bar{R},$$

statistically, no error occurs in the above extraction method. Because *L* weight parameters $w^{\prime }$ are sampled from *N* candidates in a DNN model for embedding the watermark, the above condition does not coincide with the actual robustness against a pruning attack with rate *R*.

### 3.4. Design of Two Thresholds

Because the weight of the codewords is constant, we selected α largest elements from *L* elements in the weight parameter $w^{\prime }$ extracted from the given DNN model. Although some weight parameters are cut off by the pruning attack, the values of such α elements can be retained if the threshold $T_{1}$ is appropriately designed.

Weight initialization is important to develop effective DNN models, and it is used to define the initial values for the parameters before training the models. The choice of Gaussian or uniform distribution does not have any effect, whereas the scale of the initial distribution has a significant effect on both the outcome of the optimization procedure and the ability of the network to generalize [31]. When a Gaussian distribution is selected, whose variance is studied in [32], the method is referred to as Xavier initialization. Later, [33] revealed that the Xavier initialization method does not work well with the RELU activation function, and it was revised in [34] to accommodate non-linear activation functions. These studies are based on a Gaussian assumption for the initial values, and their variance can be calculated using the libraries of PyTorch and Tensorflow.

**Figure 4:**
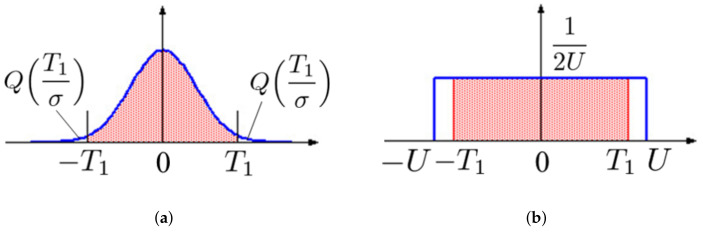
Example of probability distribution function of initial weight values (**a**) Gaussian (**b**) Uniform.

Here, we suppose that the value of weight parameters in a DNN model is modeled as a Gaussian distribution before and after the model’s training. Because the pruning attack cuts off *p* weight parameters with small values, the absolute values of α elements in $w^{†}$ should be greater than them. A statistical analysis of the distribution gives us the following inequality for the threshold $T_{1}$. Figure 4a shows the probability density function of the weight parameters. According to the figure, for a given threshold $T_{1}$, the pruning rate *R* can be calculated as

(11) $$\begin{array}{ccc} R & \leq & \frac{1}{\sqrt{2\pi \sigma^{2}}}\int_{-T_{1}}^{T_{1}}\exp\left(-\frac{x^{2}}{2\sigma^{2}}\right)dx \end{array}$$

(12) $$\begin{array}{ccc} & = & 1-\frac{2}{\sqrt{2\pi \sigma^{2}}}\int_{T_{1}}^{\infty }\exp\left(-\frac{x^{2}}{2\sigma^{2}}\right)dx \end{array}$$

(13) $$\begin{array}{ccc} & = & 1-2Q\left(\frac{T_{1}}{\sigma }\right), \end{array}$$

where Q() is the Q-function

(14) $$Q\left(t\right)=\frac{1}{\sqrt{2\pi }}\int_{t}^{\infty }\exp\left(\frac{-x^{2}}{2}\right)dx.$$

By using the inverse Q-function $Q^{-1}\left(\right)$, the appropriate threshold $T_{1}$ can be calculated for a given pruning rate $\bar{R}$.

(15) $$T_{1}=\sigma Q^{-1}\left(\frac{1-\bar{R}}{2}\right)$$

In the case of a uniform distribution in the range [−U,U], the probability density function of the weight parameters is illustrated in Figure 4b; the shaded area in the figure corresponds to the rate *R*. Here, the threshold $T_{1}$ can be calculated to satisfy the condition:

(16) $$R\leq 2T_{1}\times \frac{1}{2U}.$$

Therefore, $T_{1}$ can be given as

(17) $$T_{1}=\bar{R}U.$$

For $T_{0}$, the condition $0<T_{0}<T_{1}$ is sufficient if only a pruning attack is assumed. Considering the robustness against other attacks that modify weight parameters in a watermarked model, we should consider an appropriate margin $T_{1}-T_{0}$ by setting $T_{0}$. Setting a large value for $T_{1}$ or a small value for $T_{0}$ (a large margin of $T_{1}-T_{0}$) is ideal in terms of robustness against pruning, but it increases the amount of change in the weights. This makes abnormal features appear in the distribution of the weights, thus exposing the watermark as an attack target. On the other hand, reducing the margin of $T_{1}-T_{0}$ to prevent these features from appearing in the distribution of weights means increasing the possibility of bit-flip in the extracted codewords. Because these thresholds are a trade-off, it is necessary to set an appropriate margin of $T_{1}-T_{0}$ according to requirements.

### 3.5. Considerations

For simplicity of explanation, we assume that the weight levels are pruned in the ascending order, starting from the weight with the smallest value. The watermarking method can be extended to support more advanced pruning methods in the selection of weights, considering the pruning criteria and settings discussed in [24,25].

The usability of the proposed embedding method is confirmed from the studies of previous DNN watermarking methods. For instance, the constraint given by Equation (5) can be applied to the embedding operations in [6,7,8,9]. In the case of the method presented in [12], the embedding operation based on the constraint can be regarded as the initial assignment of weight parameters to a DNN model, and the change in weights at each epoch is corrected by iteratively performing the operation.

From the perspective of secrecy, it is better to select a small α. Attackers can use two possible approaches to cause a bit-flip in the codeword embedded into a DNN model. One approach is to identify the elements satisfying $|w_{i}|\geq T_{1}$ and decrease their weight values. Among the $N(1-\bar{R})$ candidates of weight parameters $|w_{i}|\geq T_{1}$, identifying α values becomes difficult with the increase of *N*. Because the total number of weight parameters in a DNN model is very large, executing this approach is difficult without significantly changing the weight parameters in the model. The other approach is to increase the weight values of selected weights whose values are $|w_{i}|\leq T_{0}$. Because of the large number of candidates, finding such weights without a secret key is challenging.

### 3.6. Numerical Examples

Table 1 enumerates some examples of parameters for CWC C(α,L) with respect to bit length *k* of the watermark. For instance, when a 128-bit watermark is encoded with α=20, the length of its codeword becomes L=722. Then, the code can withstand a pruning attack with a rate of R<0.9723.

If the amount of watermark information is large, it is possible to divide it into small blocks and embed each encoded block into selected weight parameters without overlapping.

## 4. Experimental Results

In this section, we encode a watermark using CWC and then embed the codeword into DNN models to evaluate the effects on DNN models. The amount of watermark is fixed to k=128 bits, and the codeword is generated by different combinations of α and *L* enumerated in Table 1. Ten different watermarks were selected from random binary sequences in this experiment. Then, the robustness against a pruning attack was measured by changing the rate *R*.

We considered the validity of the proposed method using accuracy and loss. In the case of binary classification, accuracy can be expressed as

(18) $$Accuracy_{bin}=\frac{\mathrm{TP}+\mathrm{TN}}{\mathrm{TP}+\mathrm{TN}+\mathrm{FP}+\mathrm{FN}},$$

where True Positive (TP) is a test result that correctly indicates the presence of a label, True Negative (TN) is a test result that correctly indicates the absence of a label, False Positive (FP) is a test result that wrongly indicates the presence of a particular label and False Negative (FN) is a test result that wrongly indicates the absence of a particular label, respectively. In the case of the multi-class classification used in our validation, accuracy is expressed as the average of the accuracy of each class, as shown below.

(19) $$Accuracy=\frac{\sum_{i}^{k}Accuracy_{i}}{k},$$

where, $Accuracy_{i}$ is *i*-class accuracy. And then, loss is calculated using categorical cross entropy as follows.

(20) $$Loss=-\sum_{t}\sum_{c}y_{c}^{\left(t\right)}\log{\hat{y}}_{c}^{\left(t\right)},$$

where $y_{c}^{\left(t\right)}$ is the one-hot representation of the *t*-th training data, and ${\hat{y}}_{c}^{\left(t\right)}$ is the *t*-th model output. First, we compare the accuracy of the watermarked DNN model with that of the original DNN model for a given task. Second, we run the pruning attack and check the error rate, i.e., the ratio of the number of extraction failures to the number of trials. Finally, we evaluate the error rates of the pruned DNN model after retraining.

### 4.1. Experimental Conditions

We selected the VGG16 [35] and ResNet50 [36] models as pre-trained models. These models were trained using more than 1,000,000 images from the ImageNet [37] database. A watermark was embedded into the fine-tuning model during training, similar to the experiments in [12].

#### 4.1.1. Fine-Tuning Model

Based on these pre-trained models, we fine-tuned the models with a batch size of 8 by replacing the new fully-connected (FC) layers connected to the final convolutional layer. The number of nodes at the final convolutional layer is 8192 (=4×4×512) in VGG16 and 51,200 (=5×5×2048) in ResNet50, and these nodes are connected to new FC layers with 256 nodes. The number of candidates for selecting weights from the first FC layer is more than 2,000,000, N=8192×256, in VGG16. Similarly, it is more than 13,000,000, N=51,200×256, in ResNet50. It is noted that the number *N* of weight parameters is much larger than the length *L* of the CWC codeword.

These fine-tuned models are trained using the 17 Category Flower Dataset (accessed on 25 May 2022) provided by the Visual Geometry Group of Oxford University—62.5% of images were used as training data, 12.5% as validation data, and 25.0% as test data. In this experiment, two types of fine-tuning methods were used for different purposes: fine-tuning to embed the CWC codeword and retraining to reduce the effect of pruning attacks. The epochs for fine-tuning to embed are 50 for VGG16 and 100 for ResNet50, respectively. The number of epochs for retraining the pruned models is 5.

#### 4.1.2. Threshold

The threshold $T_{1}$ must be designed appropriately to ensure robustness against pruning attacks. As discussed in Section 3.4, it depends strongly on the weight initialization. Owing to its simplicity, we selected the uniform distribution with the default setting of weight initialization [33] in the PyTorch environment.

In the VGG16-based model, we set the threshold $T_{1}$ = 0.026 for the uniform distribution in the range [−0.026650,0.026650]. This indicates that the percentage of weight parameters whose values are smaller than the threshold $T_{1}$ is 97.56%; thus, $\bar{R}=T_{1}/U=0.9756$. We also set the threshold $T_{0}$ = $T_{1}/2$ = 0.013. For the ResNet50-based model, we set the threshold $T_{1}$ = 0.010 for the uniform distribution in the range [−0.010798,0.010798]. This indicates that the percentage of weight parameters with values smaller than the threshold $T_{1}$ is 92.61%; thus, $\bar{R}=0.9261$. We also set the threshold $T_{0}$ = $T_{1}/2$ = 0.005.

### 4.2. Effect of Watermark on Original Task

When embedding a watermark into a DNN model, the accuracy of the original task should not reduce significantly. For each Hamming weight of codewords α and its length *L* by k=128 in Table 1, we compared DNN models with and without embedding in terms of accuracy and loss metrics, and the results are enumerated in Table 2, where we calculate the average of 10 trials in this experiment. Even though the results show some variation, the watermarked model does not show any noticeable difference from the original model. These results confirm that the effect of embedding a watermark into a DNN model on the performance of the original task is negligible. Although the original loss appears to be slightly higher than that of the model with embedding, this difference is within the margin of variation of the simulation.

### 4.3. Detection Performance

We have confirmed that the watermark embedded in the DNN model can be detected correctly. In the embedding procedure, weights are randomly selected from all weights in the DNN model based on a secret key. To detect the presence of hidden watermark, it is sufficient to determine whether this selected weights sequence is the CWC codeword or not.

We conducted the simulation under the setup of $\delta =0.02665,T_{1}=0.026,T_{0}=0.013$. We generated 10,000 codewords and 10,000 non-codewords, respectively, where the codewords have code length L=1757 and α=16. The non-codewords are a randomly selected sequence from a uniform distribution.

**Figure 5:**
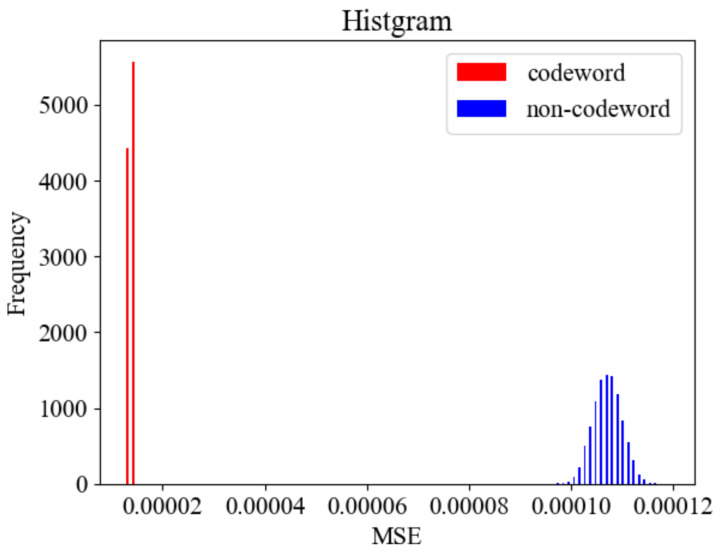
Histogram of MSEs of codeword and non-codeword.

The MSE was calculated for each of the generated codewords and non-codewords. Figure 5 shows the histogram of MSEs. As the figure shows, the distribution of MSEs can be clearly separated for codewords and non-codewords. This result implies that it is easy to distinguish codewords from non-codewords by setting a proper threshold.

### 4.4. Robustness against Pruning Attacks

We measured the robustness of the CWC codeword against a pruning attack. We selected the threshold $T_{1}$ to ensure robustness against pruning attacks with a pruning rate of $\bar{R}=0.9756$ and $\bar{R}=0.9261$ for the VGG16-based and ResNet50-based models, respectively. Unfortunately, the distribution of weight parameters changed slightly after training. Therefore, we evaluated the robustness against pruning attacks by varying *R* in the range [0,0.9] in increments of 0.1. In this evaluation, no error occurred in the extraction of the watermark. Therefore, for a detailed evaluation, we executed the pruning attack by varying the range [0.9,1.0) in increments of 0.01, whose results are shown in Table 3. The VGG16-based model has an error rate of 0% when the pruning rate is R≤0.97. The ResNet50-based model has no error for the pruning rate of R≤0.92. Thus, it is confirmed that the fine-tuned models based on VGG16 and ResNet50 are robust against pruning attacks if the pruning rate *R* is less than the designed rate $\bar{R}$, which can be determined using the CWC parameters α and *L*.

Note that robustness against a pruning attack whose pruning rate *R* is less than the designed pruning rate $\bar{R}$ is not always guaranteed, but it is statistically assured. This is because the thresholds are set from the distribution of the entire weight parameters in a DNN model, while the weights to be embedded are randomly selected from the entire weights based on the secret key. Nevertheless, under this condition, experiments show that the robustness can be well-managed by carefully selecting the thresholds $T_{1}$ and $T_{0}$.

### 4.5. Retrained DNN Model after Pruning Attack

As mentioned in Section 2.2, the DNN model is retrained after the pruning attack to recover the accuracy of the original task. We measure the accuracy for the DNN models based on VGG16 and ResNet50 before and after retraining the pruned model. Without the retraining, the higher the pruning rate, the lower the accuracy for the VGG16-based method, while the ResNet50-based model seems to be less affected by pruning attacks. Among some possible hyper-parameters, the main difference between them will be the number of weight parameters. A detailed analysis will be performed in a future work.

Table 4 shows the error rate of the CWC codeword when the pruned models are retrained. It is observed that no error occurs in the VGG16-based model when the pruning rate is R≤0.97. This indicates that the watermark becomes robust against pruning attacks if $R<\bar{R}$. In the model using ResNet50, errors still occur even when the pruning rate is R≤0.92. We speculate that this is because the weights in the FC layers of ResNet50 are more sensitive to relearning and, thus, are more likely to change. This error can be avoided by embedding watermarks in the lower layers. Although the number of trials in this experiment is small, the results confirm that the CWC can be extracted even after the pruning attack and retraining. An extensive analysis will be performed in future work. These results demonstrate that encoding using CWC guarantees the robustness of a watermarked DNN model against pruning attacks, regardless of whether it has been retrained or not.

### 4.6. Comparison with Previous Studies

Table 5 shows the effect of attacks on performance when ascending pruning attacks and random pruning attacks are performed with increasing pruning rates in previous studies [6,13,15]. In the ascending pruning attack, the top *R*% parameters are cut-off according to their absolute values in ascending, while in the random pruning attack, *R* In the evaluation of multilayer perceptron (MLP) and VGG, the bit error rate (BER) is zero up to a pruning rate of 0.9; in the evaluation of Wide ResNet (WRN), the BER is zero up to a pruning rate of 0.6 or 0.65. As discussed in Section 3.4, the robustness of our method can be controlled by defining the pair of code parameters α and *L*, and an appropriate threshold $T_{1}$, which makes our method more robust than the existing methods.

## 5. Conclusions and Future Works

We proposed a novel method to protect weight level pruning attacks in DNN watermarking by introducing the CWC. We experimentally evaluated the effect of embedding watermarks into DNN models and their robustness against pruning attacks. In addition, we evaluated the robustness of the proposed method when the DNN model is retrained after the pruning attack. We used two thresholds, $T_{1}$ and $T_{0}$, to restrict the weight parameters used to embed the watermark. Under the assumption of Gaussian or uniform distribution, $T_{1}$ can be calculated from a statistical analysis, while $T_{0}$ should be designed to consider the robustness against other possible attacks on the watermarked DNN model. However, the CWC used in our proposed study has no error-correction capability. We will consider those studies that claim CWC has error-correcting capabilities in our future work. Another future work could be designing such a model that can persist against sophisticated compressed pre-trained models.

## Figures and Tables

**Table 1:** Numerical examples of CWC parameters.

*k*	α	*L*	$\bar{R}$
64	8	972	0.9918
	9	583	0.9846
	10	393	0.9746
	11	288	0.9618
128	16	1757	0.9909
	18	1063	0.9831
	20	722	0.9723
	22	533	0.9587
256	32	3307	0.9903
	36	2011	0.9821
	40	1373	0.9709
	43	1090	0.9606
512	63	6858	0.9908
	73	3693	0.9802
	79	2780	0.9716
	85	2196	0.9613
1024	127	12,955	0.9902
	145	7443	0.9805
	159	5350	0.9703
	170	4323	0.9607

**Table 2:** Effect of embedding watermark on the original task when k=128.

(a) VGG16						
Metric	Phase	Original	C(16,1757)	C(18,1063)	C(20,722)	C(22,533)
accuracy	training	0.9639	0.9650	0.9648	0.9637	0.9634
	validation	0.9226	0.9137	0.9196	0.9238	0.9119
	test	0.9071	0.9041	0.9029	0.9088	0.9068
loss	training	0.1207	0.1147	0.1189	0.1175	0.1204
	validation	0.2288	0.2393	0.2398	0.2184	0.2575
	test	0.3703	0.3541	0.3614	0.3600	0.3662
(b) ResNet50						
Metric	Phase	Original	C(16,1757)	C(18,1063)	C(20,722)	C(22,533)
accuracy	training	0.9926	0.9924	0.9925	0.9923	0.9923
	validation	0.9310	0.9310	0.9375	0.9304	0.9405
	test	0.9288	0.9326	0.9338	0.9300	0.9382
loss	training	0.0236	0.0251	0.0238	0.0246	0.0234
	validation	0.4501	0.4099	0.4581	0.4493	0.3696
	test	0.5039	0.5558	0.5410	0.5478	0.5192

**Table 3:** Error rate against the pruning attack.

Base Model	Code	Pruning Rate (R)								
		0.91	0.92	0.93	0.94	0.95	0.96	0.97	0.98	0.99
VGG-16	C(16,1757)	0	0	0	0	0	0	0	100	100
	C(18,1063)	0	0	0	0	0	0	0	100	100
	C(20,722)	0	0	0	0	0	0	0	100	100
	C(22,533)	0	0	0	0	0	0	0	100	100
ResNet-50	C(16,1757)	0	0	100	100	100	100	100	100	100
	C(18,1063)	0	0	100	100	100	100	100	100	100
	C(20,722)	0	0	100	100	100	100	100	100	100
	C(22,533)	0	0	100	100	100	100	100	100	100

**Table 4:** Error rate against pruning attacks after retraining.

Base Model	Code	Pruning Rate (R)								
		0.91	0.92	0.93	0.94	0.95	0.96	0.97	0.98	0.99
VGG-16	C(16,1757)	0	0	0	0	0	0	10	100	100
	C(18,1063)	0	0	0	0	0	0	0	100	100
	C(20,722)	0	0	0	0	0	0	0	100	100
	C(22,533)	0	0	0	0	0	0	0	100	100
ResNet-50	C(16,1757)	90	90	100	100	100	100	100	100	100
	C(18,1063)	90	90	100	100	100	100	100	100	100
	C(20,722)	90	70	90	100	100	100	100	100	100
	C(22,533)	70	60	60	100	100	100	100	100	100

**Table 5:** Comparison of the performances of existing methods.

Baseline Model	MLP/VGG [13]	WRN [6]	WRN [15]
Ascending	0.90	0.65	-
Random	0.90	0.65	0.60

## Data Availability

The data generated and/or analyzed during the current study are available from the corresponding author on reasonable request.
